# Stress Corrosion Behaviors of 316LN Stainless Steel in a Simulated PWR Primary Water Environment

**DOI:** 10.3390/ma11091509

**Published:** 2018-08-23

**Authors:** Yong Huang, Weisong Wu, Shuo Cong, Guang Ran, Danxia Cen, Ning Li

**Affiliations:** 1 College of Energy, Xiamen University, Xiamen 361102, China; hy4480678@163.com (Y.H.); wuweisong0710@163.com (W.W.); shuocongcn@gmail.com (S.C.); 2 College of Chemistry and Chemical Engineering, Xiamen University, Xiamen 361102, China; cendanxia@163.com

**Keywords:** 316LN stainless steel, stress corrosion crack, tensile properties, nuclear materials

## Abstract

The effect of the strain rate, experimental temperature, Zn content in the test solution, and prefilming time on the mechanical properties was investigated by a tensile test with a slow strain rate, at a chemical solution of 2.2 ppm Li and 1200 ppm B in a static autoclave with 8.2 MPa. The experimental parameters clearly affected the tensile properties. The surface morphology, fractograph, and cross-sectional microstructure were analyzed by scanning electron microscopy and transmission electron microscopy. The *δ* (elongation) and UTS (ultimate tensile strength) of the samples tested in chemical solution were obviously lower than those of the samples tested under a nitrogen atmosphere. However, in general, all samples showed a ductile fracture characteristic and an excellent tensile property in all experimental conditions. The *δ* and UTS were first increased with increasing Zn content, and then decreased at both conditions of 9.26 × 10^−7^/s and 4.63 × 10^−7^/s strain rates. The difference values of tensile properties at different strain rates showed fluctuations with increasing Zn content. The *δ* increased with both increasing experimental temperature and prefilming time. The UTS first decreased with increasing prefilming time and then increased. The *I_scc_* (stress corrosion cracking susceptibility) decreased with an increasing strain rate, experiment temperature, and prefilming time. Many particles with polyhedrons were formed on the sample surfaces, which was attributed to corrosion in a periodical location at the sample surface. The average length of the particles decreased with increasing Zn content, but increased with both increasing experimental temperatures and prefilming time. The corresponding mechanism is also discussed in this work.

## 1. Introduction

316L stainless steel (316L SS) is widely used as a structural material in nuclear power plants due to its excellent mechanical properties, such as ductility, formability, toughness, and weldability, and its adequate irradiation and corrosion resistance [1,2,3]. However, in the in-service primary water environment of a watercooled reactor, 316L SS will suffer not only high temperatures and high pressure, but also stress and water chemistry corrosion. Stress corrosion cracking (SCC) [4] is a serious problem that induces the service failure of 316L SS, which is one of the greatest concerns in watercooled reactors and has become a research hotspot. Some techniques retard SCC, including mechanical surface strengthening, environmental barriers or coatings, and chemical or electrochemical corrosion potential controls such as zinc injections [5]. 

Zinc injections in a primary water environment are an effective and important method to retard the initiation of stress corrosion cracking [6,7,8], which should be attributed to anode protection, mitigation of the general corrosion rate [3], the incorporation of zinc in spinel-type oxides [8,9,10,11], and the formation of ZnCr_2_O_4_ type inner layer oxides [12,13]. Meanwhile, zinc injections could reduce radioactivity accumulation [14,15,16]. The main investigations conducted so far include the following. (1) Microstructure analysis of the surface oxide film [5,17]; (2) investigation of the kinetic and transport behaviors of surface oxidation [12]; (3) the zinc effect under different experimental conditions, including different water chemistry and loading conditions [3,18], nitrogen environments [19], irradiation [20], strain rates [21], and pH values [22]; (4) the Zn effect on the mechanical properties, such as stress corrosion crack growth rates [3]; and (5) the synergistic effect of the zinc with other injected ions on oxide films, such as aluminum [23]. The investigation of the effect of zinc content on the SCC susceptibility of Ni-base alloys [24], mill-annealed Alloy 600 [25], and Alloy 600 and 690 [26,27] by means of a slow strain rate tensile (SSRT) test was reported. Some interesting experimental results were found. For example, a > 10 ppb zinc injection reduced the SCC susceptibility. However, few reports could be found on 316LN SS. Moreover, there are still some questions associated with the mechanical properties and microstructural characteristics during the SSRT test process in chemical solutions with different zinc contents that need to be further addressed and answered, such as the effect of a previously formed oxide film in the zinc solution, and the effect of a high zinc content on the mechanical properties, the corresponding corrosion behavior, and the mechanism. The experimental temperature also affects the tensile properties, however there is not a universal law for this. For example, Jones [28] reported the tensile properties varied with experimental temperature. However, Brnic [29] reported that when the experimental temperature was increased from 250 °C to 350 °C, the mechanical properties of 316L SS almost remained constant. Wang [30] found that the corrosion rate of Alloy 690 in a primary water environment reached its maximum at 250 °C. In addition, Lee [31] developed a comprehensive SCC growth rate model and De Meo [32] reported a numerical multiphysics peridynamic framework for the modeling of adsorbed-SCC based on the adsorption induced decohesion mechanism.

Therefore, in the present work, the effect of the Zn content in the test solution, experimental temperature, strain rate, and prefilming time on the mechanical properties and microstructure during the SSRT test process was fully investigated. The surface morphology, fractograph, and cross-sectional microstructure after the SSRT test were observed and analyzed in detail by scanning electron microscopy (SEM) and transmission electron microscopy (TEM), in order to understand the corresponding mechanisms. 

## 2. Experimental Methods

316LN SS with Fe-16.96Cr-13.19Ni-2.38Mo-1.21Mn (wt %) chemical composition, which came from the components of a Pressurized Water Reactor (PWR) in China, was used in the present work. The detailed chemical composition is listed in Table 1. 

**Figure 1:**
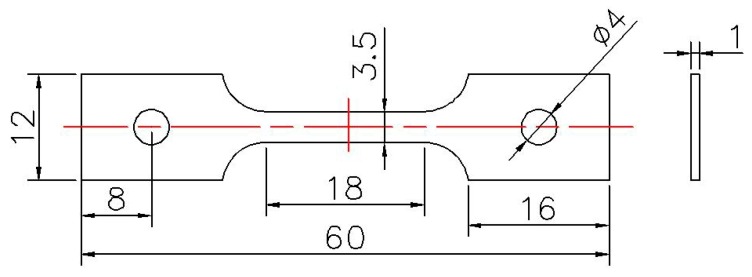
The sketch of samples used in the slow strain rate tensile (SSRT) test (Unit: mm).

The tensile samples with an 18 mm gauge length were prepared by the wire-electrode cutting method from the as-received bulk materials. The sketch of the samples for the SSRT test is shown in Figure 1. All samples first underwent grinding by a series of SiC sandpapers from #320 to #5000 grids, and were then polished by a series of diamond suspensions from 3 to 0.1 μm. Finally, the sample surface was ultrasonically cleaned in an ethanol solution and then dried in air. 

The SSRT test was performed in a static autoclave (CFS-50, Bairoe, Shanghai, China) at a high temperature and high pressure. The volume and material of the static autoclave were 4 L and 316L SS, respectively. Before the SSRT test, the autoclave interior was washed with deionized water and then with the test solution. The process was repeated three times. After that, 3.5 L of test solution was poured into the autoclave and then heated to 104 °C. Before the samples were loaded, all valves were closed. The test solution was finally heated to a designed experimental temperature, such as 300 °C. 

The experimental pressure was 8.2 ± 0.1 MPa. The deionized water, analytical-grade lithium hydroxide (LiOH·H_2_O), and boric acid (H_3_BO_3_) were proportionally mixed to form the test solution with 2.2 ppm Li and 1200 ppm B. A summary of the main experimental parameters is listed in Table 2. The main tasks of the present work were to investigate the effect of the strain rate, experimental temperature, Zn content in the test solution, and prefilming time on the tensile properties. The zinc acetate (Zn(CH_3_COO)_2_·H_2_O) was added into the test solution to form different Zn concentrations. Four groups of experiments were designed and carried out: (1) The first group of samples were tested with a Zn content from 0 to 100 ppb at a constant strain rate of 4.63 × 10^−7^/s and at 300 °C. (2) The second group of samples were tested with a Zn content from 0 to 100 ppb at a constant strain rate of 9.26 × 10^−7^/s and at 300 °C. (3) The third group of samples were tested at an experimental temperature from 250 to 330 °C at a 9.26 × 10^−7^/s strain rate and a constant chemical solution of 75 ppb Zn, 2.2 ppm Li, and 1200 ppm B. (4) The fourth group of samples were tested with a prefilming time from 0 to 600 h at 300 °C, a 4.63 × 10^−7^/s strain rate, and a chemical solution of 75 ppb Zn, 2.2 ppm Li, and 1200 ppm B. In addition, the sample #1 was tested under a nitrogen atmosphere at a 9.26 × 10^−7^/s strain rate and at 300 °C.

The surface topography and fracture morphology of the tested samples were observed by SEM on a CARL ZEISS SUPRA 55 GEMINI instrument (ZEISS, Heidenheim, Germany). In order to observe the characteristic microstructure on the sample surface, the cross-sectional TEM samples from the corrosion layer to the alloy matrix were prepared by focused ion beam (FIB) technology, covering a region that extended to areas far away from the sample surface in a field emission scanning electron microscope (FESEM)/FIB dual-beam system. The TEM observations were carried out in a JEM 2100 TEM instrument (JEOL Company, Tokyo, Japan). 

## 3. Results and Discussion

**Figure 2:**
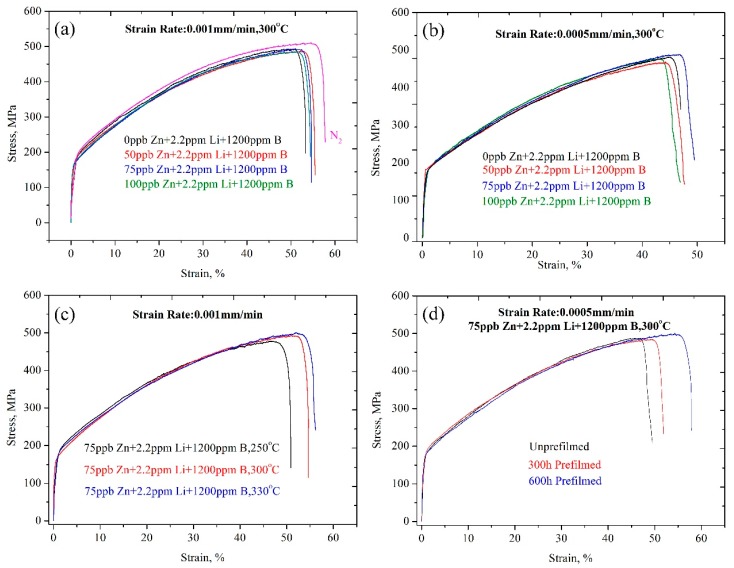
Stress–strain curves of the 316LN stainless steel tested at the conditions of (**a**) 0.001 mm/min tensile rate; (**b**) 0.0005 mm/min tensile rate; (**c**) different temperature; and (**d**) different prefilm time.

**Figure 3:**
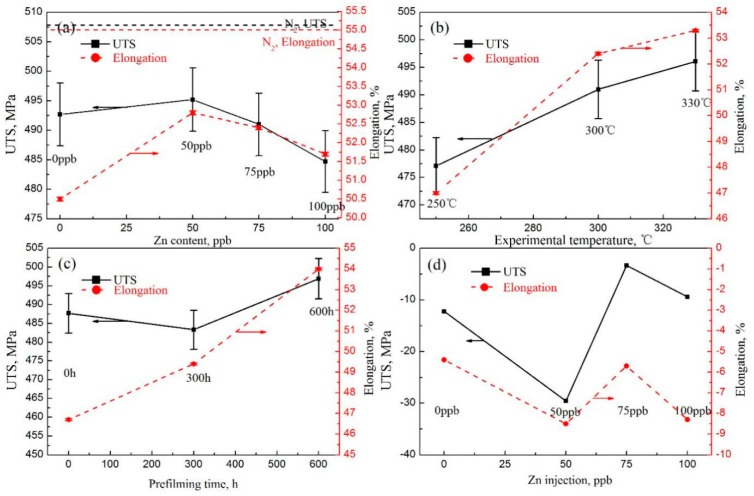
(**a**) Tensile properties vs. Zn content in the chemical solution; (**b**) tensile properties vs. experimental temperature; (**c**) tensile properties vs. prefilming time; and (**d**) reduced values of tensile properties when the strain rate decreases from 9.26 × 10^−7^/s to 4.63 × 10^−7^/s with different Zn content in chemical solution.

The stress–strain curves of the 316LN stainless steel tested at the conditions of 0.001 mm/min tensile rate, 0.0005 mm/min tensile rate, different temperature, and different prefilm time are shown in Figure 2a–d, respectively. It can be seen that the stress–strain curves of all samples show the same trend. The mechanical properties of sample #1 tested in a nitrogen environment is the best. The tensile properties of 316LN SS after the SSRT test are listed in Table 3. The shape of the stress–strain curves is similar for all samples tested in different experimental conditions, showing a ductile fracture characteristic. The relationships between the tensile properties and experimental parameters such as the strain rate, experimental temperature, Zn content in the test solution, and prefilming time are shown in Figure 3. The results indicate that the experimental parameters obviously affect the tensile properties. The *δ* (strain under maximum load) and UTS (ultimate tensile strength) of the samples tested under a nitrogen atmosphere are obviously larger than those of the samples tested in a chemical solution, as shown in Figure 3a, which indicates that chemical corrosion accelerates sample fracture and reduces the tensile properties. However, in general, the samples show excellent tensile properties under all experimental conditions. The *δ* and UTS are over 43% and 464.5 MPa, respectively.

With both a 9.26 × 10^−7^/s (0.001 mm/min) and 4.63 × 10^−7^/s (0.0005 mm/min) strain rate, the *δ* and UTS first increased with increasing Zn content, and then decreased. The *δ* and UTS are the largest in the chemical solution with 50 ppb Zn. For example, at a strain rate of 9.26 × 10^−7^/s, the *δ* and UTS are 52.8% and 495.2 MPa, respectively. The reason should be attributed to the zinc injection suppressing the hydrogen reduction reaction, forming a stable oxide film composed of a zinc–chromium phase which decreases the SCC susceptibility. As shown in Figure 3a, at the range of 0 to 50 ppb Zn in the chemical solution, both UTS and *δ* increase with increasing Zn content. The Zn oxides are cationic superchemically proportioned oxides. Zn atoms tend to occupy the tetrahedral position of AB_2_O_4_. These oxides have a normal spinel structure. With the substitution of Fe^2+^, Ni^2+^, or Co^2+^ with Zn^2+^ and the embedding of Zn^2+^ in cation vacancies, a more stable structure of ZnCr_2_O_4_ spinel is formed [33]. The newly formed compact oxide film has less cation vacancies and low solubility in water. It can prevent the oxidation of the metal atoms in the matrix and their transmigration through the oxide film. Therefore, the growth rate of the oxide film is reduced. The Zn content in the oxide film and the solution follows the thermodynamic equilibrium. The change of the oxide film after zinc injection is as follows:

$${\mathrm{Zn}}^{2+}\left(\mathrm{aq}\right)+{\mathrm{FeCr}}_{2}O_{4}\left(s\right)↔{\mathrm{Fe}}^{2+}\left(\mathrm{aq}\right)+{\mathrm{ZnCr}}_{2}O_{4}\left(s\right)$$

$${\mathrm{Zn}}^{2+}\left(\mathrm{aq}\right)+{\mathrm{NiCr}}_{2}O_{4}\left(s\right)↔{\mathrm{Ni}}^{2+}\left(\mathrm{aq}\right)+{\mathrm{ZnCr}}_{2}O_{4}\left(s\right)$$

$${\mathrm{Zn}}^{2+}\left(\mathrm{aq}\right)+{\mathrm{Fe}}_{3}O_{4}\left(s\right)↔{\mathrm{Fe}}^{2+}\left(\mathrm{aq}\right)+{\mathrm{ZnFe}}_{2}O_{4}\left(s\right)$$

The Gibbs free energy (ΔG) of ZnCr_2_O_4_ and ZnFe_2_O_4_ is −1480.415 and −1122.138 kJ/mol, respectively, while the ΔG of FeCr_2_O_4_, NiCr_2_O_4_, and Fe_3_O_4_ is −1321.836, −1398.044, and −1076.294 kJ/mol, respectively. Therefore, after the substitution of Fe^2+^, Ni^2+^, or Co^2+^ with Zn^2+^, ΔG is decreased. This means that the thermodynamic stability of the newly formed oxide film is higher [34]. Thus, the Zn injection can inhibit crack initiation and growth, increasing the mechanical property. However, beyond a certain amount of Zn injection in the chemical solution, the *δ* and UTS are decreased with increasing Zn content. The reason for this is that the thickness of the oxide film decreases rapidly as the concentration of the Zn injection is increased. Meanwhile, the crack growth rate decreases slowly. If the oxide film is too thin, it may break up earlier and the corrosion will be accelerated. 

The *δ* and UTS are increased with increasing experimental temperatures, as shown in Figure 3b. The UTS is 477.1 MPa, 491 MPa, and 496.1 MPa for temperatures of 250 °C, 300 °C, and 330 °C, respectively. The increment of UTS is 19 MPa when the temperature is increased from 250 °C to 330 °C. The *δ* is 47.0%, 52.4%, and 53.3% for temperatures of 250 °C, 300 °C, and 330 °C, respectively. It can be seen that the tensile properties are optimal at 330 °C. However, Brnic [29] reported that when the experimental temperature was increased from 250 °C to 350 °C, the mechanical properties of 316L SS almost remained constant. Wang [30] found that the corrosion rate of Alloy 690 in a primary water environment reached its maximum at 250 °C. It is known that the growth kinetics of oxide film are controlled by ion diffusion from the chemical solution to the oxidation film. The dynamic strain aging (DSA) theory proposed by Katada [35] and Atkinson [36] can be used to explain these results, in which the effect of the Zn injection on an established surface could be used to explain the environmentally sensitive fracture of the material. When the strain rate is very low, DSA more easily occurs in the range of 100 to 350 °C. 

Previous corrosion, before the SSRT test, evidently enhances the elongation, as shown in Figure 3c. The elongation is 46.7%, 49.4%, and 54% at a prefilming time of 0 h, 300 h, and 600 h, respectively. The elongation increases by about 7.3% when the prefilming time is increased from 0 h to 600 h. However, the UTS is first decreased with increasing prefilming time, and then increased. The UTS is about 496.9 MPa after previous corrosion for 600 h. In all, previous corrosion improves the tensile properties. Prefilming can increase the specific gravity of Cr oxide and Zn oxide in the surface oxide film, and can inhibit the initiation and propagation of surface cracks, which can improve the mechanical properties of the material and reduce the sensitivity of stress corrosion. The mechanical properties of 316LN SS with prefilming for 600 h are obviously greater than those without prefilming, and are the opposite for the susceptibility to stress corrosion. Liu [17] found that the initial water chemistry played a key role for the characteristics of surface oxide films. The films become much more compact with increasing exposure time in the Zn-injection solution.

Figure 3d shows the relationship between the difference values of the tensile properties at the strain rate of 9.26 × 10^−7^/s and 4.63 × 10^−7^/s, and Zn content in the chemical solution. The difference values of the tensile properties decrease with increasing Zn content, then increase and finally decrease again. The UTS and *δ* at a strain rate of 9.26 × 10^−7^/s are larger than those at a strain rate of 4.63 × 10^−7^/s. The reason is due to the chemical corrosion being much more serious, and decreasing the tensile properties at a strain rate of 4.63 × 10^−7^/s. The lower the strain rate is, the longer the corrosion time will be.

**Figure 4:**
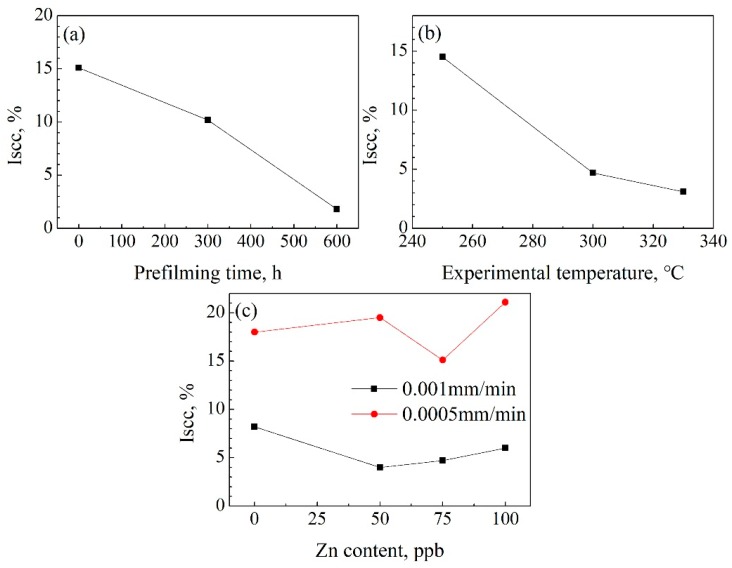
(**a**) *I_scc_* vs. prefilming time; (**b**) *I_scc_* vs. the experimental temperature; and (**c**) *I_scc_* vs. Zn content in the chemical solution.

*I_scc_* (stress corrosion cracking susceptibility, *I_scc_*), calculated according to Equation (1), can be used to evaluate the SCC susceptibility of 316LN SS [37]. The larger the *I_scc_* value is, the more sensitive to SCC it will be. The results show that previous corrosion in a chemical solution with 75 ppb Zn content clearly reduces the SCC susceptibility, as shown in Figure 4a. The *I_scc_* is 15.1, 10.2, and 1.8 at a prefilming time of 0 h, 300 h, and 600 h, respectively. The *I_scc_* decreases with increasing experimental temperatures, as shown in Figure 4b. The *I_scc_* is 14.5, 4.7, and 3.1 for temperatures of 250 °C, 300 °C, and 330 °C, respectively. However, as shown in Figure 4c, the lower the strain rate is, the larger the *I_scc_* value will be.

(1) $$I_{scc}=1-\frac{\delta_{f}^{x}}{\delta_{f}^{N_{2}}}$$

where, $\delta_{f}^{N_{2}}$ and $\delta_{f}^{x}$ are the elongation after the SSRT test in a nitrogen atmosphere and under special experimental conditions, respectively.

**Figure 5:**
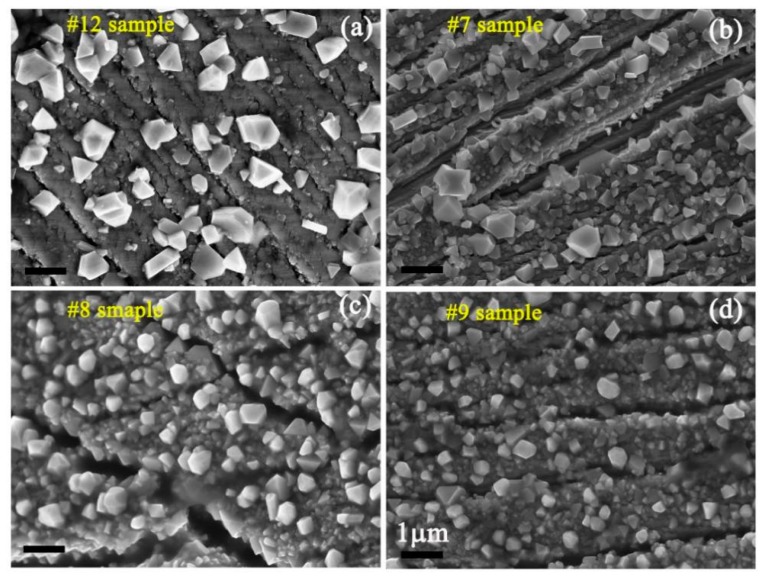
SEM images showing the surface morphologies of (**a**) #12 sample; (**b**) #7 sample; (**c**) #8 sample; and (**d**) #9 sample after the SSRT test.

Figure 5 presents SEM images showing the surface morphologies of the samples after the SSRT test at four kinds of experimental conditions. Some characteristic particles with polyhedrons are formed on the samples’ surfaces. The formed particles should be spinel oxides, such as FeCr_2_O_4_, ZnFe_2_O_4_, and ZnCr_2_O_4_. It is known that Fe_3_O_4_ and FeCr_2_O_4_ are common corrosion products of stainless steel [38,39]. With the addition of Zn in the chemical solution, ZnFe_2_O_4_ and ZnCr_2_O_4_ could be formed in the oxide film [17,40]. 

**Figure 6:**
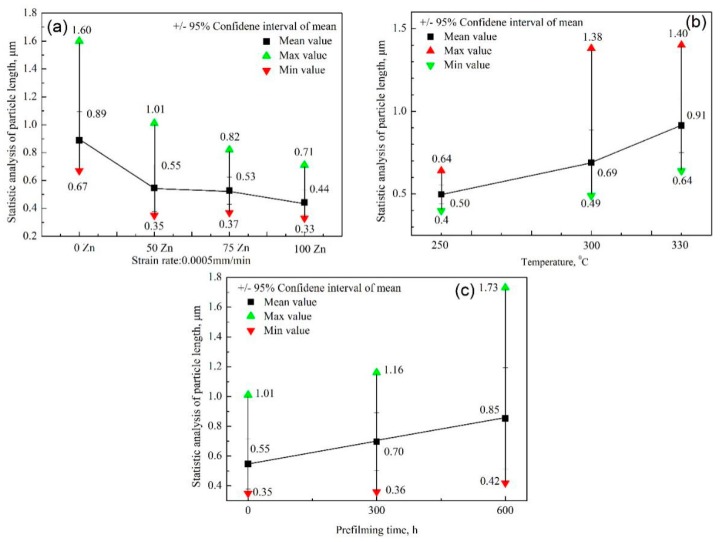
Statistical analysis results of the particles on the sample surface: (**a**) Length vs. Zn content; (**b**) length vs. experiment temperature; and (**c**) length vs. prefilming time.

In order to investigate the variation tendency of the average length of the formed particles with experiment parameters, the species of the formed particles on the sample surface do not be distinguished. The results of the statistical analysis of the average length of the formed particles on the sample surface are shown in Figure 6. Quantitative analysis of particle size was conducted using a Nano Measure software (Nano Measure 1.2.5, Shanghai, China). The counting numbers for one sample exceeded 100. The error bar was less than 1%. The results show that the average length of the formed particles decreased with increasing Zn content at a 4.63 × 10^−7^/s strain rate. The average length is about 0.89 μm and 0.44 μm with chemical solutions of 0 ppb Zn and 100 ppb Zn, respectively. The average length increased with increasing experiment temperatures, which can be attributed to the high chemical activity at a high experiment temperature, and the corresponding high corrosion rate and high migration rate of matrix atoms. The average length is about 0.5 μm, 0.69 μm, and 0.91 μm at 250 °C, 300 °C, and 330 °C, respectively. It can be seen that the average length is increased by about two times when the experiment temperature is increased from 250 °C to 330 °C. The longer the previous corrosion time was, the larger the average length of the particles will be. The average length is up to 0.85 μm with previous corrosion of 600 h, which is about 1.55 times that with nonprevious corrosion. 

**Figure 7:**
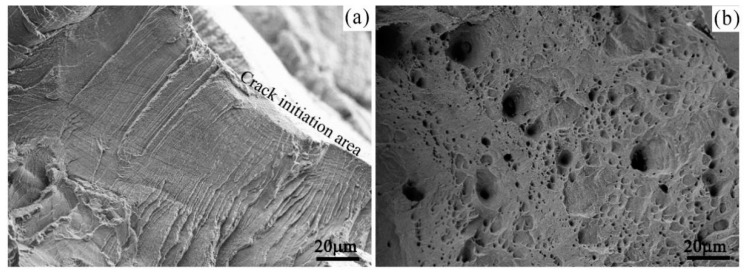
SEM images showing the fracture structure: (**a**) Crack initiation area; (**b**) dimples.

The SEM fractographs of the samples tested under the experimental condition of previously corrosion for 300 h, with the experiment solution of 75 ppb Zn, 2.2 ppm Li, and 1200 ppm B are shown in Figure 7. In general, the studied alloy shows a clear ductile fracture nature. The crack initiates from the sample surface, as denoted in Figure 7a, and the secondary cracks are also seen in the fracture surface. According to the morphology, the fracture surface has a pseudo-cleavage feature and plastic tearing feature (a large number of dimples as shown in Figure 7b) in the last stage of crack growth at a high magnification. The fracture feature and fractograph are similar for the samples tested under all experimental conditions.

**Figure 8:**
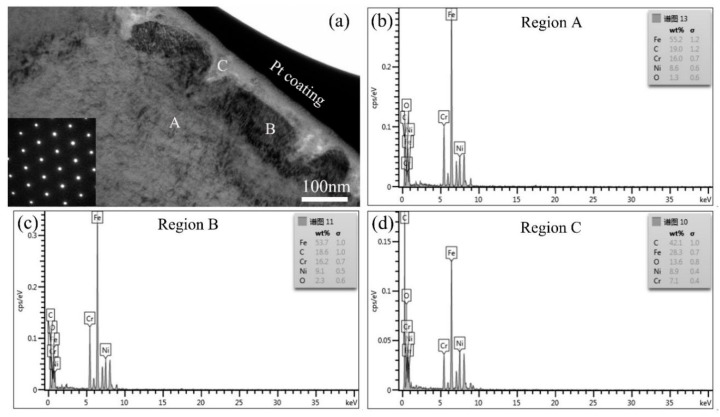
(**a**) Cross-sectional bright field TEM image of sample #12; (**b**–**d**) EDS results of locations ‘A’, ‘B’, and ‘C’ in (**a**), respectively.

In order to observe the characteristic microstructure and analyze the formation mechanism of the particles on the sample surface, the TEM sample, which was perpendicular to the surface of tensile sample #12, was prepared by in situ focused ion beam. The Pt coating as indicated in Figure 8a was used to protect the microstructure during TEM preparation. The cross-sectional bright field TEM image is shown in Figure 8a. Grains with a rectangular shape, as denoted by the letter ‘B’, can be observed at the sample subsurface. An oxidation corrosion layer covers the rectangular grains. The inserted image located at the left-bottom corner of Figure 8a is the corresponding selected area electron diffraction pattern at location ‘B’, which shows the austenite crystal structure. The energy dispersive spectrum (EDS) analysis results of locations ‘A’, ‘B’, and ‘C’ in Figure 8a are shown in Figure 8b–d, respectively. The semiquantitative results of the analysis elements are shown in Table 4. It can be seen that the content of elemental O and C in the location ‘C’ is much higher than in the locations ‘A’ and ‘B’. The opposite is true for elemental Fe and Cr. However, the content of elemental Ni is similar in all locations. In addition, compared with test results in location ‘A’, the elemental content in location ‘B’ is the same if the measurement error by the EDS method is taken into account. Therefore, location ‘B’ also belongs to the alloy matrix. Similar results are obtained from several other locations. In fact, the content of elemental Zn is very low, and thus it is difficult to detect by EDS technology. 

From the cross-sectional morphology of the oxidation layer, an interesting phenomenon of the periodical vertical-depth corrosion, as indicated by the letter ‘C’, is observed. Therefore, the corrosion in a periodical location at the sample surface induces the formation of particles with a polyhedron shape on the sample surface. The reference reported the resistance to corrosion mainly related to the formation of a chromium-rich passivation film in the corrosive environment [41]. However, in the present work, the oxide film has a low Cr content and high O content compared with the original alloy matrix, especially for the C element. The phases in the oxide film should be the mixtures of carbides and oxides. However, the excessive content of the C element in the oxide film was not found in previous research.

## 4. Conclusions

The effect of the strain rate, experimental temperature, Zn content in the test solution, and prefilming time on the tensile properties was investigated by a tensile test with a slow strain rate, with a chemical solution of 2.2 ppm Li and 1200 ppm B in a static autoclave at 8.2 MPa. The surface morphology, fractograph, and cross-sectional microstructure were analyzed by SEM and TEM. The main conclusions are as follows:

(1) The experimental parameters obviously affected the tensile properties. The *δ* and UTS of the samples tested under a nitrogen atmosphere were clearly larger than those of the samples tested in the chemical solution. However, in general, all samples showed a ductile fracture characteristic and an excellent tensile property under all experimental conditions.
(2) The *δ* and UTS first increased with increasing Zn content, and then decreased at both a 9.26 × 10^−7^/s and 4.63 × 10^−7^/s strain rate. The *δ* and UTS were largest in the chemical solution with 50 ppb Zn. The difference values of the tensile properties at different strain rates showed fluctuations with increasing Zn content. The lower the strain rate was, the larger the *I_scc_* value would be.
(3) The *δ* and UTS increased with increasing experimental temperature. The UTS was 477.1 MPa, 491 MPa, and 496.1 MPa for temperatures of 250 °C, 300 °C, and 330 °C, respectively. Correspondingly, the *δ* was 47.0%, 52.4%, and 53.3%, respectively. The *I_scc_* decreased with increasing experimental temperatures.
(4) The previous corrosion before the SSRT test evidently enhanced the elongation. The elongation was 46.7%, 49.4%, and 54% at a prefilming time of 0 h, 300 h, and 600 h, respectively. However, the UTS first decreased with increasing prefilming time, and then increased. The UTS was about 496.9 MPa after previous corrosion for 600 h. Previous corrosion in the chemical solution obviously reduced the SCC susceptibility.
(5) Many particles with a polyhedron shape were formed on the sample surfaces, which was attributed to corrosion in a periodical location at the sample surface. The average length of the formed particles decreased with increasing Zn content, but increased with increasing experimental temperatures. The longer the previous corrosion time was, the larger the average length of particles would be.

## Figures and Tables

**Table 1:** Chemical composition of 316LN SS used in the present work (mass fraction, %).

Element	C	Si	Mn	P	S	Cr	Ni	Mo	N	Co	Fe
Content	0.019	0.22	1.21	0.014	0.002	16.96	13.19	2.38	0.14	0.012	Bal.

**Table 2:** Main experimental parameters during the slow strain rate tensile (SSRT) test.

Samples	#1 *	#2	#3	#4	#5	#6	#7	#8	#9	#10	#11	#12	#13
Zn content (ppb)	0	0	50	75	100	0	50	75	100	75	75	75	75
Experiment temperature	300 °C									250	330	300 °C	
Strain rate	9.26 × 10^−7^/s					4.63 × 10^−7^/s				9.26 × 10^−7^/s		4.63 × 10^−7^/s	
Prefilming time, h	0	0	0	0	0	0	0	0	0	0	0	300	600

* #1 sample was tested under a nitrogen atmosphere.

**Table 3:** Tensile properties of 316LN SS after the SSRT test.

Sample	#1	#2	#3	#4	#5	#6	#7	#8	#9	#10	#11	#12	#13
UTS, MPa	507.3 ± 5.48	492.7 ± 5.32	495.2 ± 5.35	491 ± 5.30	484.7 ± 5.23	480.5 ± 5.19	464.6 ± 5.01	487.7 ± 5.26	475.3 ± 5.13	477.1 ± 5.15	496.1 ± 5.35	483.3 ± 5.22	496.9 ± 5.36
*δ*, *%*	55.0 ± 0.05	50.5 ± 0.05	52.8 ± 0.05	52.4 ± 0.05	51.7 ± 0.05	45.1 ± 0.05	44.3 ± 0.05	46.7 ± 0.05	43.4 ± 0.05	47.0 ± 0.05	53.3 ± 0.05	49.4 ± 0.05	54 ± 0.05
*I_scc_*, %	/	8.2	4.0	4.7	6.0	18.0	19.5	15.1	21.1	14.5	3.1	10.2	1.8

**Table 4:** The percentage content of the main elements in Figure 8a, wt %.

Main Elements	Fe	Cr	Ni	C	O
Location ‘A’	55.2	16.0	8.6	19.0	1.3
Location ‘B’	53.7	16.2	9.1	18.6	2.3
Location ‘C’	28.3	7.1	8.9	42.1	13.6

## References

[1] Nezakat M., Akhiani H., Penttilä S., Sabet S.M., Szpunar J. (2015). Effect of thermo-mechanical processing on oxidation of austenitic stainless steel 316L in supercritical water. Corros. Sci..

[2] Was G.S., Ampornrat P., Gupta G., Teysseyre S., West E.A., Allen T.R., Sridharan K., Tan L., Chen Y., Ren X. (2007). Corrosion and stress corrosion cracking in supercritical water. J. Nucl. Mater..

[3] Zhang L., Chen K., Wang J., Guo X., Du D., Andresen P.L. (2017). Effects of zinc injection on stress corrosion cracking of cold worked, austenitic stainless steel in high-temperature water environments. Scr. Mater..

[4] Terachi T., Yamada T., Miyamoto T., Arioka K. (2012). SCC growth behaviors of austenitic stainless steels in simulated PWR primary water. J. Nucl. Mater..

[5] Liu X., Wu X., Han E.H. (2011). Influence of Zn injection on characteristics of oxide film on 304 stainless steel in borated and lithiated high temperature water. Corros. Sci..

[6] Betova I., Bojinov M., Kinnunen P., Saario T. (2011). Zn injection in Pressurized Water Reactors–laboratory tests, field experience and modelling. Research Report No. VTT-R-05511-11.

[7] Marks C., Dumouchel M., Reid R., White G. (2011). Quantifying the benefit of chemical mitigation of PWSCC via zinc addition or hydrogen optimization. Proceedings of the 15th International Conference on Environmental Degradation of Materials in Nuclear Power System-Water Reactors.

[8] Norring K., Engström J. (2008). Initiation of SCC in nickel base alloys in primary PWR environment: Studies at Studsvik since mid 1980s. Energy Mater..

[9] Piippo J., Saario T., Tegeder V., Stellwag B. (1996). Influence of zinc on properties and growth of oxide layers in simulated primary coolant. Proceedings of the 7th International Conference on the Water Chemistry of Nuclear Reactor Systems.

[10] Ziemniak S.E., Hanson M. (2006). Zinc treatment effects on corrosion behavior of Alloy 600 in high temperature, hydrogenated water. Corros. Sci..

[11] Liu X., Wu X., Han E.H. (2013). Electrochemical and surface analytical investigation of the effects of Zn concentrations on characteristics of oxide films on 304 stainless steel in borated and lithiated high temperature water. Electrochim. Acta.

[12] Betova I., Bojinov M., Kinnunen P., Lundgren K., Saario T. (2009). Influence of Zn on the oxide layer on AISI 316L (NG) stainless steel in simulated pressurized water reactor coolant. Electrochim. Acta.

[13] Huang J., Liu X., Han E.H., Wu X. (2011). Influence of Zn on oxide films on Alloy 690 in borated and lithiated high temperature water. Corros. Sci..

[14] Kim Y.J., Andresen P.L. (2003). Transformation kinetics of oxide formed on noble metal-treated type 304 stainless steel in 288 °C water. Corrosion.

[15] Pathania R.S., Cheng B.O. (1998). Evaluation of zinc addition to the primary coolant of Farley-2 PWR. Nucl. Power Plant.

[16] Hanzawa Y., Hiroishi D., Matsuura C., Ishigure K. (1998). Solubility of zinc ferrite in high-temperature oxygenated water. J. Nucl. Mater..

[17] Liu X., Wu X., Han E.H. (2012). Effect of Zn injection on established surface oxide films on 316 L stainless steel in borated and lithiated high temperature water. Corros. Sci..

[18] Lu Z., Shoji T., Meng F., Qiu Y., Dan T., Xue H. (2011). Effects of water chemistry and loading conditions on stress corrosion cracking of cold-rolled 316NG stainless steel in high temperature water. Corros. Sci..

[19] Toppo A., Pujar M.G., Mallika C., Mudali U.K., Dayal R.K. (2015). Effect of nitrogen on stress corrosion behavior of austenitic stainless steels using electrochemical noise technique. J. Mater. Eng. Perform..

[20] Furutani G., Nakajima N., Konishi T., Kodama M. (2001). Stress corrosion cracking on irradiated 316SS. J. Nucl. Mater..

[21] Totsuka N., Nishikawa Y., Kaneshima Y. (2005). Effect of strain rate on primary water stress corrosion cracking fracture mode and crack growth rate of nickel alloy and austenitic stainless steel. Corrosion.

[22] Liu X., Han E.H., Wu X. (2014). Effects of pH value on characteristics of oxide films on 316L stainless steel in Zn-injected borated and lithiated high temperature water. Corros. Sci..

[23] Zhang S., Shi R., Chen Y., Wang M. (2018). Corrosion behavior of oxide films on AISI 316L SS formed in high temperature water with simultaneous injection of zinc and aluminum. J. Alloy. Compd..

[24] Andresen P.L., Wilson J.A., Ahluwalia K.S. Use of primary water chemistry in PWRs to mitigate PWSCC of Ni-Base alloys. Presented at the International Conference on Water Chemistry of Nuclear Reactor Systems.

[25] Kawamura H., Hirano H., Shirai S., Takamatsu H., Matsunaga T., Yamaoka K., Oshinden K., Takiguchi H. (2000). Inhibitory effect of zinc addition to high-Temperature hydrogenated water on mill-annealed and prefilmed Alloy 600. Corrosion.

[26] Angell M.G., Allan S.J., Airey G.P. (1999). The effect of primary coolant zinc additions on the SCC behavior of alloy 600 and 690. Proceedings of the 9th International Conference on Environmental Degradation of Materials in Nuclear Power System—Water Reactors.

[27] Maeng W.Y., Cho Y.S., Kim U.C. Effect of Zn injection on the SCC crack growth of Alloy 600 in water at 360 °C. Presented at the International Conference on Water Chemistry of Nuclear Reactor Systems.

[28] Jones R.H., Henager C.H. (1992). Effect of gamma irradiation on stress corrosion behavior of austenitic stainless steel under ITER-relevant conditions. J. Nucl. Mater..

[29] Brnic J., Niu J., Canadija M., Turkalj G., Lanc D. (2009). Behavior of AISI 316L steel subjected to uniaxial state of stress at elevated temperatures. J. Mater. Sci. Technol..

[30] Wang J., Wang J., Ming H., Zhang Z., Han E.H. (2017). Effect of temperature on corrosion behavior of Alloy 690 in high temperature hydrogenated water. J. Mater. Sci. Technol..

[31] Lee D., Huang Y., Achenbach J.D. (2015). A comprehensive analysis of the growth rate of stress corrosion cracks. Proc. R. Soc. A.

[32] Wang J., Wang J., Ming H., Zhang Z., Han E.H. (2016). Modelling of stress-corrosion cracking by using peridynamics. Int. J. Hydrogen Energy.

[33] Lister D.H. (1993). Activity transport and corrosion processes in PWRs. Nucl. Energy.

[34] Ziemniak S.E., Hanson M. (2006). Corrosion behavior of NiCrFe alloy 600 in high temperature, hydrogenated water. Corros. Sci..

[35] Katada Y., Nagata N. (1985). The effect of temperature on fatigue crack growth behaviour of a low alloy pressure vessel steel in a simulated BWR environment. Corros. Sci..

[36] Atkinson J.D., Yu J. (1997). The role of dynamic strain-ageing in the environment assisted cracking observed in pressure vessel steels. Fatigue Fract. Eng. Mater. Struct..

[37] Tang Z., Hu S., Zhang P. (2012). Stress corrosion cracking of 316Ti in 300 °C high temperature water containing chloride ions. J. Chin. Soc. Corros. Prot..

[38] Kim Y.J. (1995). Characterization of the oxide film formed on type 316 stainless steel in 288 °C water in cyclic normal and hydrogen water chemistries. Corrosion.

[39] Kim Y.J. (1999). Analysis of oxide film formed on type 304 stainless steel in 288 °C water containing oxygen, hydrogen, and hydrogen peroxide. Corrosion.

[40] Ziemniak S.E., Hanson M. (2006). Zinc treatment effects on corrosion behavior of 304 stainless steel in high temperature, hydrogenated water. Corros. Sci..

[41] Ehrnstén U. (2012). Corrosion and stress corrosion cracking of austenitic stainless steels. Compr. Nucl. Mater..

